# A new method using Raman spectroscopy for *in vivo* targeted brain cancer tissue biopsy

**DOI:** 10.1038/s41598-018-20233-3

**Published:** 2018-01-29

**Authors:** Joannie Desroches, Michael Jermyn, Michael Pinto, Fabien Picot, Marie-Andrée Tremblay, Sami Obaid, Eric Marple, Kirk Urmey, Dominique Trudel, Gilles Soulez, Marie-Christine Guiot, Brian C. Wilson, Kevin Petrecca, Frédéric Leblond

**Affiliations:** 1 Dept. of Engineering Physics, Polytechnique Montreal, CP 6079, Succ. Centre-Ville, Montreal, QC H3C 3A7 Canada; 2 Centre de Recherche du Centre Hospitalier de l’Université de Montréal, 900 rue, Saint-Denis, H2X 0A9 QC Canada; 3 Thayer School of Engineering, Dartmouth College, 14 Engineering Dr, Hanover, NH 03755 USA; 4 Brain Tumour Research Centre, Montreal Neurological Institute and Hospital, Dept. of Neurology and Neurosurgery, McGill University, 3801 University St., Montreal, QC H3A 2B4 Canada; 5 Division of Neurosurgery, Hôpital Notre-Dame du CHUM, University of Montreal, Montreal, 1560 Sherbrooke E, Montreal, QC H2L 4M1 Canada; 6 EMVision LLC, 1471 F Road, Loxahatchee, Florida 33470 United States; 7 Department of Pathology, Centre Hospitalier Universitaire de Montréal, 1058 Rue Saint-Denis, Montreal, Québec, H2X 3J4 Canada; 8 Centre hospitalier de L’Université de Montréal, Hôpital Notre-Dame-Pavillon Lachapelle, Montréal, QC H2L 4M1 Canada; 9 Division of Neuropathology, Department of Pathology, Montreal Neurological Institute and Hospital, McGill University, 3801 University St, Montreal, QC H3A 2B4 Canada; 10 University Health Network/University of Toronto, TMDT 15-314, 101 College St., Toronto, ON M5G 1L7 Canada

## Abstract

Modern cancer diagnosis requires histological, molecular, and genomic tumor analyses. Tumor sampling is often achieved using a targeted needle biopsy approach. Targeting errors and cancer heterogeneity causing inaccurate sampling are important limitations of this *blind* technique leading to non-diagnostic or poor quality samples, and the need for repeated biopsies pose elevated patient risk. An optical technology that can analyze the molecular nature of the tissue prior to harvesting could improve cancer targeting and mitigate patient risk. Here we report on the design, development, and validation of an *in situ* intraoperative, label-free, cancer detection system based on high wavenumber Raman spectroscopy. This optical detection device was engineered into a commercially available biopsy system allowing tumor analysis prior to tissue harvesting without disrupting workflow. Using a dual validation approach we show that high wavenumber Raman spectroscopy can detect human dense cancer with >60% cancer cells *in situ* during surgery with a sensitivity and specificity of 80% and 90%, respectively. We also demonstrate for the first time the use of this system in a swine brain biopsy model. These studies set the stage for the clinical translation of this optical molecular imaging method for high yield and safe targeted biopsy.

## Introduction

Most solid cancers are discovered by conventional imaging techniques such as computed tomography (CT), magnetic resonance imaging (MRI), positron emission tomography (PET), and ultrasound (US). While these images characterize the location(s) and anatomical relationships of the cancer, most of them have limitations in terms of tumor grading and molecular characterization. Consequently, direct sampling for histological, molecular, and genomic characterisation is required for diagnosis and treatment planning. The quality of the tissue obtained for analysis is thus of vital importance to guide the clinical trajectory. Furthermore, as targeted therapies are known to cause imaging changes that appear to represent cancer progression, but are in fact only imaging changes caused by the treatments, oncology teams require high quality tumor sampling during and post-treatment in order to make correct treatment decisions^1^. As the use of these new therapies becomes more common the need for new and more informed tissue sampling approaches are needed.

To aid tumor targeting, biopsies are performed using approaches guided by conventional imaging such as US, CT, and MRI. Since these imaging techniques do not provide the user information regarding the nature of the tissue prior to harvesting, and the precision of lesion targeting may be compromised by tissue shifts between the time of the imaging session and procedure or during the procedure, poor quality sampling often occurs. For example, the prostate cancer detection rate is only 40–45% at initial biopsy^2–4^. Similarly, the diagnostic yield of lung cancer biopsies varies from 42 to 100%. Importantly, non-diagnostic samples in brain cancer biopsies leading to repeat procedure occurs in up to 24% of cases^5–7^, and diagnostic errors still occur in 10–30% of cases^8^. For each pass of a biopsy needle there is an associated risk of hemorrhage and infection that can negatively affect patient outcomes^6,9–12^.

Current approaches to reduce the risk of poor quality sampling include intraoperative sample analysis based on histological processing such as tissue smears and frozen tissue sections, or harvesting multiple samples to increase the likelihood of a diagnostic sample. These approaches are resource intensive as they require 20–30 minutes for each sample to be processed and analysed thereby lengthening the duration of the procedure. Moreover, this analysis does not provide molecular information. The addition of real time molecular tissue characterization capabilities to guide biopsy needle insertion and tissue collection can enhance the information provided to surgeons and interventionalists prior to sampling thereby improving the rate of high quality diagnostic yields. Informed tissue sampling also reduces patient risk by limiting the number of samples collected and reducing the rate of repeat procedures.

A strategy for real time informed cancer needle biopsy is to interrogate the tissue using optical techniques *in situ* prior to collection. This interrogation can provide the user with online feedback regarding the molecular nature of the tissue to confirm the targeting site, characterise the presence of cancer, avoid injury to major blood vessels, and avoid sampling necrotic tissue or tumor regions that do not represent the pathology. Optical techniques have been developed for this purpose, including the use of the metabolic fluorescent biomarker protoporphyrin (Pp) IX that is endogenously synthesized in tumor cells following administration of 5-aminolevulenic acid. The red PpIX fluorescence could then be used to guide the insertion of a needle biopsy, as demonstrated clinically in brain on *ex vivo* biopsy tissue and *in vivo* in mouse models^13–15^. However, the technique requires the use of a contrast agent, which presents issues in terms of clinical practicality and tumor sensitivity. Optical coherence tomography (OCT) is also investigated for the guidance of needle biopsies, for applications in breast, prostate and lung cancer^16–20^. Recently, a RS probe that fits inside the bore of a hypodermic needle was developed for lymph-node biopsy guidance^21,22^. Although these are impressive advances, OCT has yet to show it’s ability to detect cancer *in vivo*.

In the past our group has developed Raman spectroscopy instruments for surgical guidance, demonstrating *in vivo* fingerprint (FP) Raman Spectroscopy (RS) can distinguish between normal brain, tumor tissue and necrosis with accuracies upwards of 90% in patients for grade 2–4 gliomas or metastasis associated with lung, colon and skin cancer cells^23–28^. The majority of biomedical RS studies have used near-infrared illumination and spectral detection in the 500–2000 cm^−1^ range^29–33^, commonly referred to as the *fingerprint* (FP) region, since it is rich in biochemical information associated with vibrational bonds of proteins, lipids, nucleic acids and amino acids. The high wavenumber (HWN) region, from about 2000 to 4000 cm^−1^, contains information relating to *e.g*. CH-, OH- and NH- stretching modes. High wavenumber shifts have been found to discriminate different types of brain tissue *ex vivo*, including cortex and white matter^34,35^. Stimulated Raman scattering (SRS) microscopy in the HWN regime has been used to differentiate tumors from normal brain *in vivo* in mice bearing intracranial glioblastoma xenografts^27^.

Here, we are presenting the development and preliminary validation of a new integrated biopsy system that provides *in situ* cancer characterization using spontaneous Raman spectroscopy. The device is in the form of an integrated Raman probe, engineered into a commercial biopsy system, with minimal disruption to the surgical workflow. First, a small-scale animal study is presented demonstrating that the novel optical needle can measure deep brain Raman spectra and collect tissue samples at the same location. Secondly, an *in vivo* human validation study is presented to demonstrate -during brain cancer resection surgeries- that a HWN RS intraoperative probe can accurately detect cancer tissue *in situ*. This sets the stage for the clinical translation of this optical molecular imaging method for high quality and safe targeted cancer biopsy.

## Results

### Core needle biopsy system for interstitial vibrational spectroscopy

We have developed an instrument that can provide, rapidly and with minimal impact on the flow of the clinical procedure, interstitial (*in situ*) label-free vibrational spectroscopy of tissue to ensure biopsy samples are collected in a location containing densities of cancer cells sufficiently high for reliable diagnosis. Despite the richer molecular information content associated with FP region, the HWN region was chosen since it allowed the use of a practical fiberoptic configuration that satisfies the stringent miniaturization constraints required for direct integration with a commercial brain biopsy needle. In particular, fiberoptic FP probes typically require interference filters to be positioned close to the distal end of the fibers to minimize detection of the background due to elastic (Rayleigh) light scattering in the tissue and the contribution of Raman-active molecules from the optical fibers^36^. This is crucial for intraoperative applications because of the rarity of inelastic scattering events such that real time (<1 s) detection can only be achieved in situations where the contribution of non tissue-specific backgrounds is minimal. However, the *filter constraint* is relaxed in the HWN region because of the low silica Raman activity in the fibers and the large shift between the excitation and the detection spectral bands that markedly reduces the elastic scattering contribution to the detected signal.

**Figure 1:**
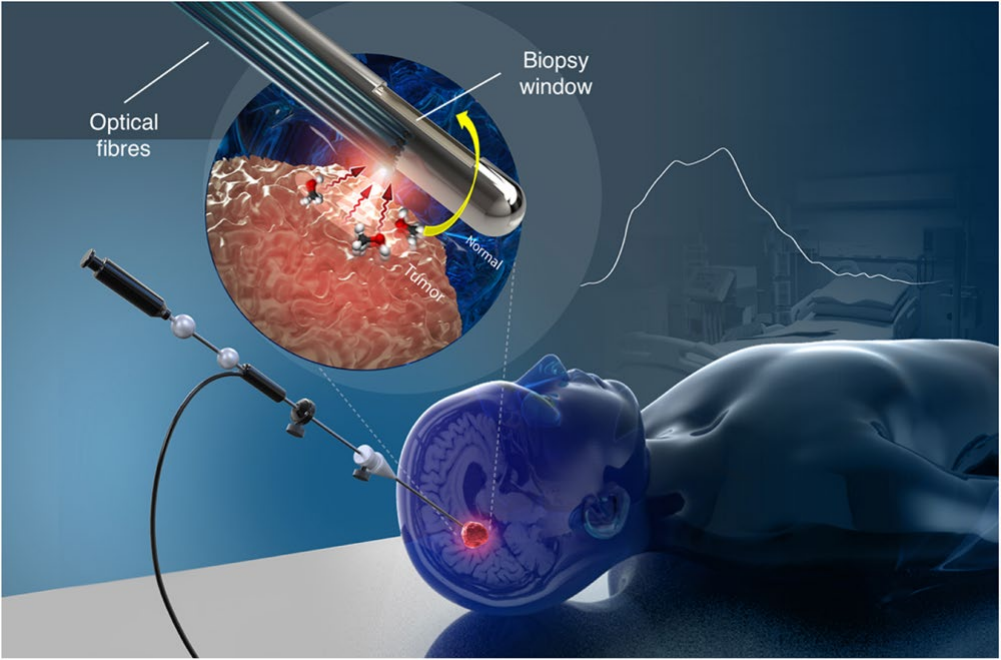
Schematic representation of the optical core needle biopsy, with a magnified view of the tip showing the biopsy window and the beveled optical fibers used for illumination and detection. The fibers are located opposite to the biopsy window and the needle is rotated by exactly 180° to collect a tissue sample spatially co-localized with a spectral measurement. Figure produced by Dariush Bagheri.

A core needle biopsy consists of an outer cannula and an inner cannula, both with rectangular windows through which a tissue sample can be aspired and cut following rapid relative rotation of the two cannulas. Optical measurement capability was integrated into a commercial brain needle biopsy (Medtronic, Inc) by attaching optical fibers around the periphery of the outer cannula (Fig. 1). The distal tips of these fibers were polished at an angle for side illumination and positioned to ensure that measurements are made in the middle (along the main axis) of the biopsy window. The collected tissue spectra correspond spatially to the physical tissue biopsy volume once the operator rotates the needle by 180° prior to sample collection. A biocompatible polymer cover with low Raman signal (no carbon-hydrogen bonds) protected the optical fibers. For initial proof-of-principle *in vivo* measurements, one of the optical fibers was connected to a 671 nm diode laser for excitation and the reemitted light was detected through the same fiber by a spectrometer (ANDOR Technologies, Inc). Instrument control and data acquisition were achieved using a custom LabView software (National Instruments, Inc), allowing the Raman spectra to be visualized in real time as they are collected. Each spectrum covered the range 2600–3800 cm^−1^ at a spectral resolution ∼1.8.cm^−1^. More details relating to the design and fabrication of the interstitial optical system are provided in the *Methods* section.

### Interstitial Raman spectroscopy measurements

**Figure 2:**
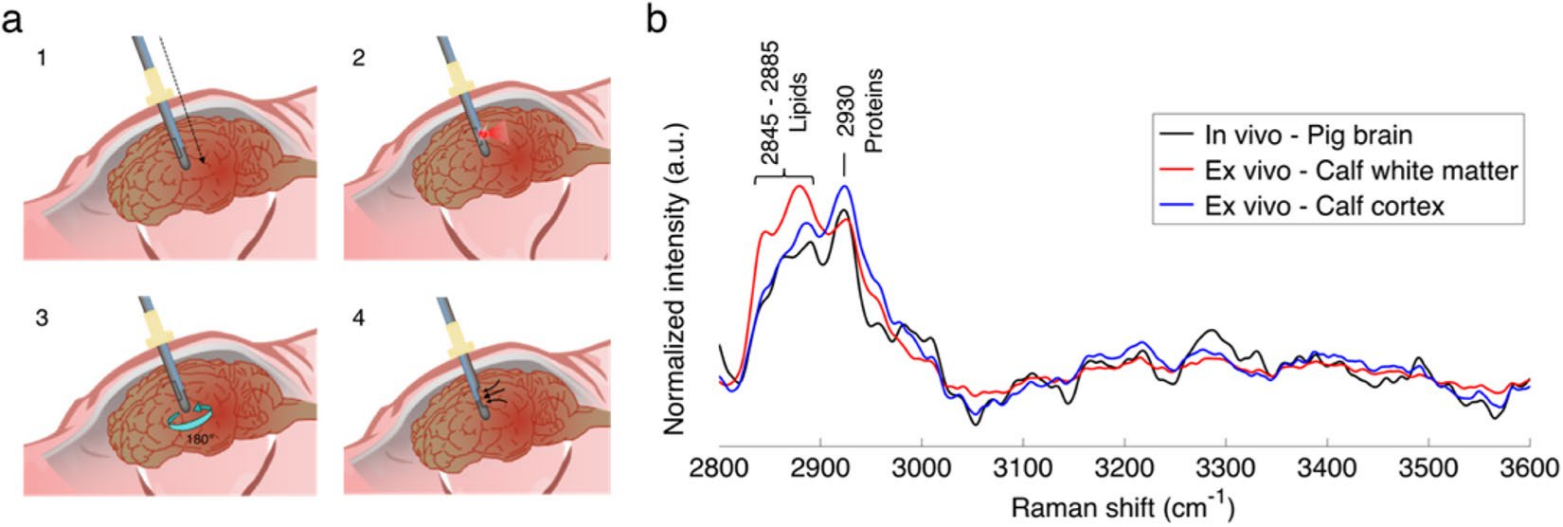
RS measurements in normal brain. (**a**) Schematic of the *in vivo* acquisition steps: 1) needle insertion along the planned trajectory, 2) RS collection, 3) 180° rotation of the needle, and 4) tissue collection. (**b**) *In vivo* Raman spectra averaged over all measurements (*n* = 11) and compared with *ex vivo* spectra for white matter and cortex.

Measurements were made to insure that the optical needle biopsy system can retrieve reliable HWN Raman spectra in brain tissue. Initially, *ex vivo* spectra were acquired on a calf brain to evaluate the ability of the system to distinguish between white and gray matter. The tip of the excitation/detection fiber was placed in contact with either white or gray matter at 20 different locations and the mean spectra were computed for each tissue type (Fig. 2b). Compared with gray matter (blue spectrum), the white matter spectrum (in red) demonstrates larger contributions from lipids (2845 cm^−1^) and a lower contribution from proteins and nucleic acids (2930 cm^−1^), in agreement with the literature^26,34^.

An experiment was then conducted in two pig brains *in situ* to demonstrate technical feasibility of deep brain spectroscopy. Computed tomography angiography (CTA) of the animal head was done before the procedure to determine a safe trajectory for the needle in order to avoid major blood vessels, and a frameless system (Medtronic, Inc.) was used to secure the needle on the cranium and to ensure a straight trajectory into the brain is followed (Fig. 2a). After intracranial insertion of the needle through a burr hole, Raman measurements were made at several locations (21 in total) along its trajectory. After each measurement, the needle was rotated by 180° and a tissue sample was collected for histopathology analysis. Of the 21 brain samples, 9 were heterogeneous (containing both white matter and cortex), 8 contained only cortex, 1 contained only white matter and 3 contained other brain structures such as blood vessels. The mean spectrum associated with samples containing cortex and/or white matter (n = 18) was computed (Fig. 2b). The resulting *in situ* brain spectrum is consistent with the *ex vivo* data, showing characteristic spectral features of cerebral tissue, mostly from proteins (2930 cm^−1^) and lipids (2845 to 2885 cm^−1^). This experiment confirmed that the device can be easily and safely inserted into the brain and generate Raman spectra with low acquisition time (Table S2) and minimal disruption of a standard biopsy procedure.

### Intraoperative Raman spectroscopy measurements during human glioma surgery

**Figure 3:**
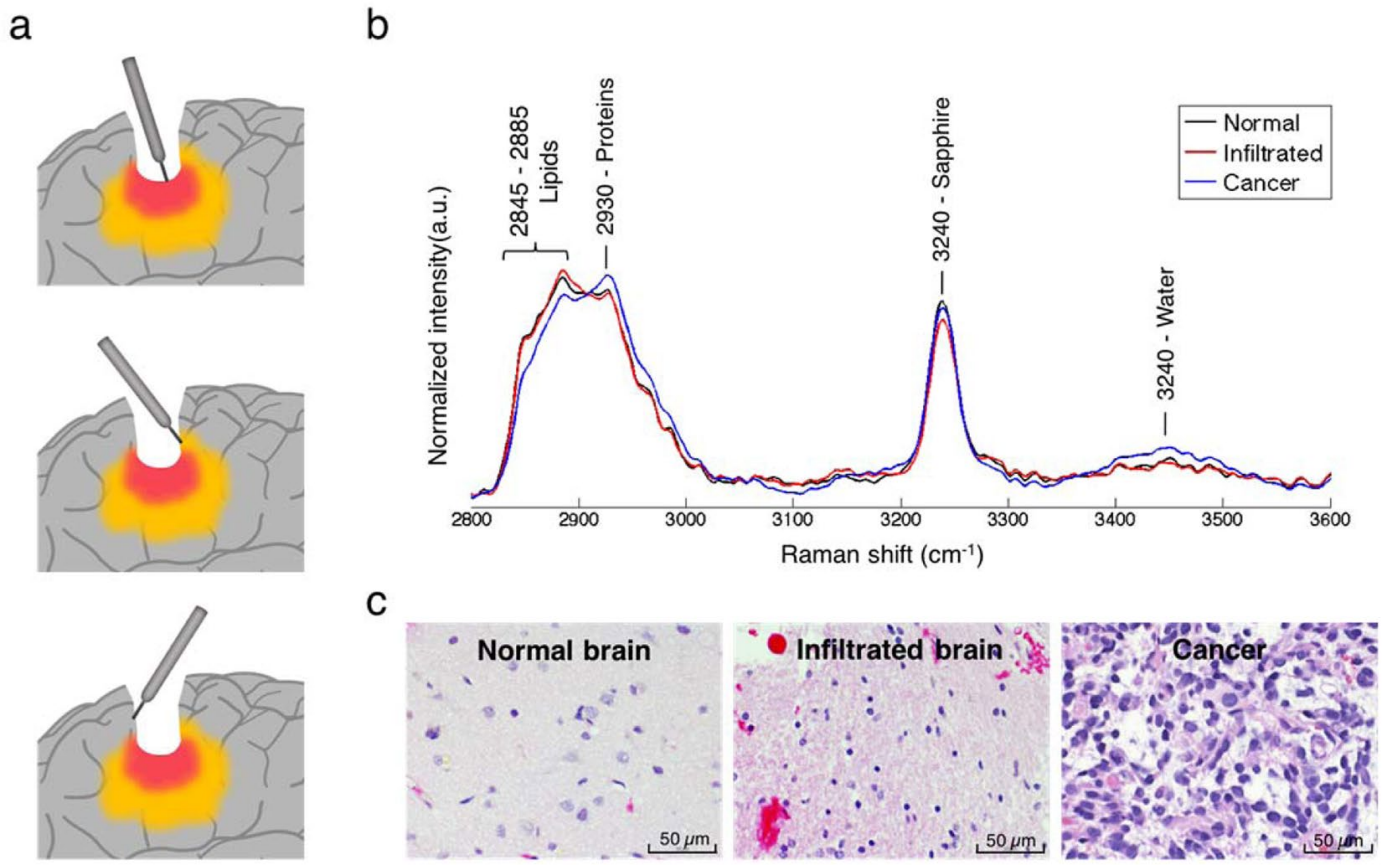
(**a**) Schematic depiction of *in vivo* RS measurements taken in the surgical cavity during glioma resection using the handheld contact probe in dense cancer (red), infiltrated brain (yellow) and surrounding normal brain. (**b**) *In vivo* high wavenumber Raman spectra of dense cancer, infiltrated brain and normal brain, averaged over all samples. (**c**) Representative H&E-stained micrographs for each tissue type.

A clinical study was conducted in 19 grade 2–4 glioma patients to determine whether or not HWN RS can differentiate normal brain from tissue containing cancer cells. The system setup and clinical validation details can be found in the *Methods* section, together with further details relating to the intraoperative use of the hand-held contact probe that we have reported previously in which the FP rather than the HWN region was used^23^. Ten to fifteen sites were interrogated in each patient for a total of 280 measurements (Fig. 3a). Tissue was sampled at each site for blinded histopathological analysis to estimate the percentage of cancer cells within the interrogated region based on Hematoxylin & Eosin (H&E) staining (see *Methods* for details). Information relating to sample size and patient histological diagnosis information is given in Table 1.

**Table 1:** Patient histological diagnosis, indicating tumor grade and type as well as sample size information.

		*n* patients	*n* samples *(n normals)*
Age (year), median (range)	54 (31–77)		
WHO grade	Grade 2		
	Astrocytoma	2	20 *(6)*
	Oligodendroma	1	22 *(4)*
	Grade 3		
	Oligodendroglioma	2	35 *(16)*
	Astrocytoma	1	15 *(8)*
	Grade 4		
	Glioblastoma	13	188 *(71)*
Tissue type	Normal brain		105
	Dense cancer		124
	Infiltrated		51
Total		19	280

In total, 105 measurements were made on *normal brain* (no cancer cells present), 51 in *infiltrated brain* (≤60% cancer cells present), and 124 in *dense cancer* (>60% cancer cells present). In the case of infiltrated brain tissue, the percentage of cancer cells in the samples ranged from only a few infiltrative cells (<5%) to 60%. The mean spectra for *normal brain*, *infiltrated brain* and *dense cancer* for all patients were computed (Fig. 3c). Sample histopathology images for the three groups are provided showing H&E images of dense cancer, infiltrated brain and normal brain (Fig. 3c). The *in vivo* spectra for normal brain tissue acquired with the hand-held HWN RS probe are consistent with the spectra acquired in calf and swine brain using the optical biopsy needle.

### High wavenumber Raman spectroscopy can differentiate cancer from normal brain

**Figure 4:**
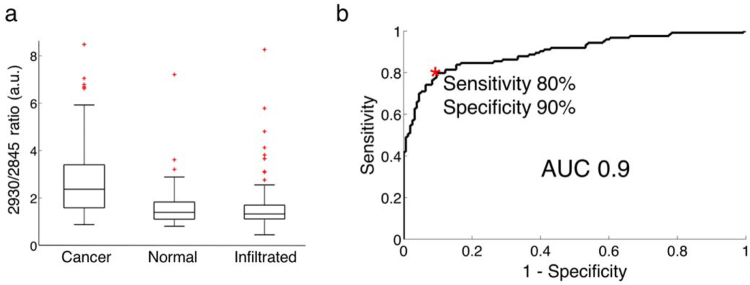
(**a**) Boxplots of the ratio of the lipid and protein bands (2930 cm^−1^/2845 cm^−1^) for normal brain, infiltrated brain and dense cancer tissue in glioma patients. (**b**) Receiver operating characteristic (ROC) curve computed using the SVM algorithm and leave-one-out cross-validation. The indicated point at the minimal distance from the upper-left corner of the ROC curve was chosen for calculating the sensitivity and specificity values.

Identified prominent peaks (Fig. 3b) are associated mainly with CH_2_ symmetric and asymmetric stretches of lipids and proteins (2845–2885 cm^−1^), symmetric CH_3_ stretch due primarily to proteins (2930 cm^−1^) and the OH stretching from water molecules (at 3450 cm^−1^). The peak at 3240 cm^−1^ comes from the sapphire of the tip lens in the hand-held contact probe. It was not observed in the spectra acquired with the optical biopsy needle since no lens was used in the design of that instrument. The most significant biochemical differences between *dense cancer* and *normal brain* were assessed based on a two-sided *t*-test of the individual RS peaks. Tissue bands associated with some biomolecules are dominant either in *normal brain* or *dense cancer* (Table S1). Particularly, peaks associated mostly with the presence of proteins (2930 cm^−1^) were found to be higher for *dense cancer* tissue. Figure 4a shows box plots of the ratio of inelastic scattering intensity at 2930 and 2845 cm^−1^, a metric used to quantify the relative fraction of proteins and lipids. These data demonstrate an increase in the protein/lipid ratio for *dense cancer* compared to *normal brain* samples, consistent with findings by Ji *et al*. in *ex vivo* brain samples using stimulated RS^27^. However, when compared with that of normal brain, the protein/lipid ratio in *infiltrated* samples does not show significant differences.

While individual measures such as peak ratios can provide valuable biochemical information for tissue differentiation, only multivariate approaches are able to fully utilize the spectrally distributed HWN RS information. Classification of the data was therefore performed using a support vector machine (SVM) algorithm and its performance was preliminarily evaluated using a leave-one-out cross-validation (LOOCV) approach, with the histopathological assessment as ground truth. The most important clinical goal here is to follow needle trajectory planning using conventional imaging methods by a confirmation that the needle has reached a location associated with a brain area containing cancer cells in proportions relative to normal brain than enable reliable diagnosis. Here, the tumor cells density that was deemed sufficient was set at 60% based on neuropathologist experience (more details in the *Methods* section). Therefore, the unmet clinical need to reduce non-diagnostic biopsies can be addressed if the proposed technique can detect – and thus target for collection – cancer tissue associated with a large enough density of cancer cells. For classification purposes the 280 samples were divided into two categories. The so-called *non-diagnostic* category contains samples that should not have been collected because they are either associated with normal brain or with infiltrated tissue containing less than 60% of cancer cells. The second category, labeled *dense cancer* is associated with samples with >60% cancer cells and that should have been collected for diagnostic purposes. Based on multivariate classification, *dense cancer* samples could be distinguished from non-diagnostic samples with an accuracy of 84%, a sensitivity of 80%, a specificity of 90% and a receiver-operating-characteristic ROC curve with an area-under-the-curve (AUC) of 0.9 (Fig. 4b).

## Discussion

There is a critical need in modern medicine for new molecular targeting techniques that can be seamlessly integrated with standard biopsy procedures to increase diagnostic yield and insure optimal treatment planning. Here, using brain cancer as a model, we developed a *real time* interstitial tissue characterization technique acquiring molecular vibrational spectroscopy measurements at the distal end of a commercial biopsy needle to ensure co-location with biopsy sample collection. A detailed proof of principle was presented, initially demonstrating the new optical biopsy needle can detect subtle molecular differences between white matter and cortex *ex vivo* in calf brain, followed by a technical feasibility of *in vivo* spectroscopy and tissue sampling deep into the brain in a swine model. Then, using a different instrument (an intraoperative handheld probe) during 19 human neurosurgical resection procedures, we provided evidence that the same molecular contrast detected by the new system could be used to locate cancer tissue *in vivo* (sensitivity and specificity >80%) to target biopsy location. Because the optical fibres are integrated with a commercial biopsy needle with measurements taking <2 s, the new optical biopsy needle could be used as a synergistic complement to standard of care brain biopsy procedures with minimal disruption of the clinical workflow. Less constraining strategies, in which placement of miniaturized optical probes within the outer cannula of the needle alternates with placement of the inner cannula for tissue collection, were not considered because of the potential to add significant time to the clinical workflow and so limit the number of measurements that can be made. Clinical translation of the proposed technique could eliminate the need for inserting a separate optical probe and collecting multiple samples as well as reduce repeat brain needle biopsy procedures by insuring *only one* biopsy sample is harvested that contains a sufficient density of cancer cells for diagnosis. Incidentally this could allow optimal treatment planning as well as lead to a reduction of the risks to patient health caused by haemorrhages and infections during needle insertion and tissue collection.

A crucial component of future in-human clinical studies with the new system will be the development of robust statistical models insuring cancer cells detection with supervised multivariate techniques can be achieved *in vivo* during a procedure^37^. However, this requires the acquisition of large patient datasets, which could be challenging in terms of achieving statistical significance and capturing the full heterogeneity associated with tumors and normal brain, given only a few biopsy samples are normally collected per biopsy procedure^37^. An indirect but nevertheless valid approach is to train the statistical model on vibrational spectroscopy data acquired *in vivo* during surgical resection, since multiple measurements/samples can then be acquired for each patient. This is precisely what was done here using the intraoperative handheld probe^23,24^ during open-cranium surgery. A critical factor insuring the validity of this approach will be to take into account any differences in the instrument spectral-response function to allow the algorithm trained with the intraoperative handheld probe to be directly applied to data acquired with the optical biopsy needle. In the present study the observed differences between brain spectra acquired with the optical needle (Fig. 2) and the intraoperative probe (Fig. 3) are attributable to differences in the noise level, and the contribution from the Raman-active molecules in the contact probe lens. The photonic noise is larger in the optical needle spectra, since it uses only a single optical fibre with a smaller diameter, whereas the contact probe uses 7 collection fibres of larger core diameter (Table S2). Additionally, the effective excitation power is lower by a factor of ∼5 in the optical needle. These signal discrepancies will be offset by using all 12 needle fibers simultaneously at comparable excitation powers and signal integration times, and applying separately-measured corrections for the different spectral-response functions. Importantly, the statistical models will be made using data where the spectral region around the Raman peak associated with the lens material is truncated to ensure exact correspondence between data acquired with the different probes. In this proof-of-concept study, a single fiber (out of 12 that were available) was used for excitation and detection. However, several fibers could have been use in spatial offset mode^38–40^ to increase detection volume and penetration depth. In the case of cancer cells detection, considering that the signal of interest is very weak, there is a compromise between detection volume and signal to noise ratio. Decreasing detection volume might produce a better sensitivity for detection, but only if the signal to noise ratio remains unchanged. This translates to a higher laser power or acquisition time, so less clinical practicality. An interesting study would be to determine the optimal combination of detection volume, laser power and integration time to lead the best sensitivity for a specific application.

The results of the present experiments set the stage for a clinical study using the optical biopsy needle to provide *live* tissue classification during brain needle biopsy procedures. However, brain tissue sampling is among the most accurate biopsy procedures, in large part due to the use of stereotactic approaches and advanced image-guided techniques. Online molecular characterization would be even more important for diagnostic of disease in other organ systems where conventional imaging techniques are less specific and guidance techniques used less accurate^41^. Oncology applications with high levels of misdiagnosis at biopsy include prostate and lung, but also other organ sites such as breast and colon^2,10–12,42–45^. Because of all the tissue processing steps needed before microscopic histopathological analyses, the presence of cancer in the biopsy cores is only confirmed after a few days, and up to several weeks if a molecular analysis is required. In cases of uncertain diagnosis repeat biopsies are required, adding significant delay before diagnosis can be obtained and a treatment chosen, and increasing risk for the patient. The new technique also shows great potential in interventional medicine applications beyond biopsies to optimize targeted treatment methods such as in laser-induced thermotherapy (LIIT)^46^, brachytherapy^47,48^ or therapeutics injection of viruses into tumors.

Although clinical translation of the new technique for organ systems other than brain has the potential to significantly reduce instances of misdiagnosis, it will require significant efforts to insure the optical technology is integrated into clinical practice in a manner that is minimally disruptive to the standard workflow. This will not only include developing the vibrational spectroscopy technique to fit with instrumentation used as the standard of care (*e.g*., bronchoscope, laparoscope) but will also require the development of statistical models for each pathology of interest. Here, we have presented a proof of principle demonstrating clinical integration of high wavenumber Raman spectroscopy in brain needle biopsy procedures. Although more challenging from an engineering perspective, developing optical biopsy needles detecting in the fingerprint region would lend even more molecular information, with incidental increases in sensitivity and specificity for cancer detection^23,24,28^ and thus a potential for even more precise targeting for biopsy sample collection.

## Methods

### Core needle biopsy system design and fabrication

The biopsy needle probe comprises 12 low-hydroxyl fibers of 105 µm core diameter, 150 µm outer diameter and 0.22 NA (numerical aperture) for excitation and collection, distributed around the needle to provide 200° azimuthal angular coverage. Since total internal reflection is used to create a side viewing fiber, an air interface is required at the angle polished end of the fiber. The fiber tips were polished at a 37° angle to ensure total internal reflection. 12 thin polymer tubes (170 µm inner diameter, 355 µm outer diameter) were heat fused together, and each of their distal ends were heat fused close to ensure no liquid infiltration. The 12 tubes were placed around the external cannula of the needle, on the 200° not covered by the biopsy window. The fibers were inserted in each tube one at a time so that the angled end is aligned to the center of the needle biopsy window, and rotated to the proper orientation to ensure the fiber emission and collection cone was normal (outward) to the BNB needle. This is repeated for all 12 fibers. The tubes around the fibers create the air interface at the angled ends, required for total internal reflection. Epoxy was used to close the proximal tube ends to prevent any liquid infiltration. A thin wall heat shrink was placed over both ends of the tubes/fibers to hold them in place on the needle. The heat shrink was not placed over the angled portion of the fibers so no Raman signature of the heat shrink would be collected. The fibers at the proximal end of the needle were placed in a 1 m long nylon protective tube (1.55 mm inner diameter, 1.8 mm outer diameter) to a breakout section which each fiber is protected individually by a 0.75 m long nylon protective tube (1.55 mm inner diameter, 1.8 mm outer diameter). The fibers were terminated individually with FC/PC connectors.

### Ex vivo tissue measurements

For the current proof-of-principle experiments, a single fiber of the needle probe was used. A 785 nm dichroic beam splitter (Semrock, Inc) is used to separate the illumination and detection light and the light collection used a laser-line band-pass filter (R_avg_ > 97% from 705–900 nm, Semrock, Inc) and a long-pass filter (T_avg_ > 93% from 812.9–1200 nm, Semrock, Inc), together with collimating lenses (Thorlabs, Inc). A 750 mW, 671 nm spectrum stabilized near-infrared laser (Laser Quantum, Inc) with adjustable power is used for Raman excitation. The collection path is connected to a spectrograph comprising a diffraction grating coupled to a high-resolution charge-coupled-device (CCD) camera (ANDOR Technology, Inc.). A fresh normal calf brain was used for the *ex vivo* proof-of-concept study, since this has a Raman signature and background similar to that of human brain tissue^24^. Contact Raman spectral measurements were made on different locations, and tissues were labeled *white matter* or *gray matter* by visual assessment. An imaging sequence comprises a background acquisition (laser turned off) and acquisition of 3 Raman spectra at the same location, with integration times of 0.5–2 s for each measurement. A fixed laser power of 10 mW was used to avoid damaging the polymer tubing from blood heating around the fiber tips. All ambient sources of light were turned off during acquisitions.

### *In vivo* measurements in swine brain

The *in vivo* animal study was approved by the institutional Ethics Review Board (CHUM Research Center, Montreal, Canada), and all methods were performed in accordance with the relevant guidelines and regulations. Two adult 20–30 kg pigs were anaesthetised using a standard injection and gas inhalation procedure. Raman measurements were acquired using two different procedures in each animal (through a burr hole and following craniotomy) to access the brain with the RS biopsy needle. In both cases an X-ray contrast agent (Visipaque 370, 15 ml) was first injected via a catheter placed in the carotid artery, and CT difference angiography was performed to highlight intracranial blood vessels. For the first animal, the burr hole location was selected and a trajectory for the needle was chosen to achieve maximum depth and avoid intercepting major blood vessels during needle insertion through the dura. The Navigus frameless system (Medtronic, Inc.) was installed on the skull and the biopsy needle was aligned to its calculated trajectory. The needle was inserted until it reached the target location using navigational software (iGuide, Siemens Healthcare, Inc.). Data acquisition consisted of a 0.5 s background measurement (laser off) and 3 Raman spectra measurements with 0.5 s acquisition time at the target location. The probe was then rotated 180° and a tissue sample (typically 1 cm long and ∼1–2 mm wide) was extracted by opening the biopsy window, aspirating tissue into the inner cannula and cutting the sample by rotating the inner cannula. These acquisition steps were repeated at different insertion depths (n = 8) while pulling out the needle along its trajectory. The samples were fixed in formalin for subsequent histopathologic assessment to distinguish gray and white matter. In the second pig, a craniotomy was performed to provide direct access to the brain. Since finding locations of white and gray matter is difficult based on a single trajectory of the needle, this approach was used on the second animal to access different areas of the brain more easily. The needle was inserted manually into the brain, and the RS and biopsy sequence was repeated for various locations and depths (n = 13). The total time for optical measurements and tissue sample collection was ∼15–20 min for each animal. The measured spectra were processed as follows: 1) averaging of the 3 spectra at each location and background subtraction (measurement with the laser off), 2) correction for the instrument response using measurements of a calibrated halogen white light (National Institute of Standards and Technology, Maryland, USA), 3) removal of intrinsic tissue fluorescence using an iterative polynomial fit^49^ and 4) standard normal variate (SNV) normalization. Spectra from samples found on pathology to include other tissues such as blood vessel and choroid plexus were excluded from the analysis, so that 18 spectra were kept for the analysis and their average was computed.

### High wavenumber Raman spectroscopy with intraoperative probe

*In vivo* validation was performed with a contact intraoperative probe that was previously used to demonstrate that fingerprint RS is able to distinguish normal brain tissue from tissue containing cancer cells^23,24^. In order to use the same spectrometer (same spectral range in nanometers), a 671 nm laser was employed with this probe to measure higher frequency shifts. Slight modifications were also made to the design of the intraoperative probe previously used to make it capable of both fingerprint and high wavenumber RS. This modified probe has a 2.1 mm outer diameter containing 7 low-hydroxyl, 300 µm core fibers for light collection distributed around a central 272 µm core source fiber. The reemitted signal passes through a ring-shaped notch filter to remove elastically backscattered light and light scattered from the lens surface. The band-pass filter used in front of the excitation fiber was replaced with an in-line short-pass filter to allow the 671 nm excitation. A custom sapphire 2-component lens was used at the tip of the probe to overlap the excitation and detection volumes. The illumination and detection instruments are the same as for the animal study, previously described in detail. The 7 collection fibers were connected to the spectrometer through a single custom-built SMA connector. The laser spot size of the probe (tissue excitation area) of the handheld probe had a diameter of 0.5 mm and a field of view associated with a single point of that same diameter. Further, light transport simulations in tissue using a Monte Carlo technique demonstrated that the sampling depth is ∼ 1mm^50^. Similar results are expected both for the handheld probe and the optical biopsy needle since tissue excitation and detection are spatially co-located.

### Validation during brain tumor resection

This study investigated high-wavenumber RS using the modified handheld contact probe in 19 adult patients undergoing open cranium surgery for grade 2–4 glioma at the Montreal Neurological Institute and Hospital, under institutional Ethics Board approval and with informed consent. All methods were performed in accordance with the relevant guidelines and regulations. Preoperative neurological examination was performed, as well as standard clinical imaging by magnetic resonance (MRI). For the intraoperative measurements the system was placed on a surgical cart, brought in the operating room and connected to the previously sterilized probe. Purpose-built acquisition software was operated by a member of the instrument-development group, while the neurosurgeon handled the probe within the surgical sterile area. The surgeon first put the probe in contact with brain tissue in the surgical cavity followed by Raman spectroscopy acquisition. The RS sequence comprised a series of single-point spectral acquisitions with step-wise automatic laser power adjustment to find a power that optimized the use of the detector’s dynamic range without saturation, a background acquisition with the laser turned off, and then collection of 3 Raman spectra. Each spectral measurement required that the neurosurgical microscope light be turned off temporarily to minimize ambient-light artefacts^24^. Following spectral acquisition, the neurosurgeon collected a small biopsy sample (~1.5 mm) at the interrogated site, located by the visible indentation of the tissue surface from the probe contact. Biopsy samples were fixed in formalin, embedded in paraffin, H&E stained and evaluated using a standard blinded protocol by an expert neuropathologist. Using standard clinical practice, atypical cells were identified based on their morphological features, including nuclear atypia and nuclear polymorphism and mitotic figures. Each sample was classified as either: *normal brain* (no cancer cells present), *infiltrated* (presence of cancer cells within non tumoral brain parenchyma), or *dense cancer*. For each infiltrated sample, the pathologist estimated the percentage of cancer cells in the sample (number of cancer cells divided by the total number of cells). Samples were rejected if optical artifacts were observed in the Raman signal (4 samples), if the CCD saturated during acquisition (3 samples) or if the biopsy sample was partitioned, i.e. containing focal area(s) of different tissue types (10 samples).

### Data analysis and tissue classification

Processing for RS involves background subtraction, correction for the instrument response, the removal of intrinsic tissue fluorescence^49^ and normalization. Regarding classification, classes were chosen based on the clinical application and limitations of brain needle biopsy. The density of cancer cells in the sample plays an essential role in obtaining all the components for the integrated diagnosis of gliomas, as recommended by the WHO classification of tumors of the central nervous system^51^. Analysis of samples containing a minimum number of tumor cells is required for the identification of biomarkers essential for accurate diagnosis, ensuring optimal treatment planning and prognosis for the patient. For example, standard of care for the management of patients with glioma includes the detection of the IDH1 R132H point mutation as well as the status of the promoter of the MGMT gene for which, as is also the case for DNA sequencing, a minimum percentage of tumoral cells is necessary to get reliable and reproducible results^52,53^. Thus, a threshold of 60% of cancer cells or higher was set since it is sufficient to achieve a precise diagnosis, based on neuropathologist experience (MCG). A Support Vector Machine (SVM) technique was used for RS tissue classification using 141 features of the spectra, chosen based on the *p*-values computed for the correlation between a particular spectral feature and the tissue type. Therefore, the spectral features used were those most correlated with the difference between cancer and normal tissue. The number of features was optimized to find the best outcome. Leave-one-out cross-validation (LOOCV) was used to determine the classification accuracy, sensitivity and specificity, treating all spectra except one as the training data for the classifier, with the other spectra as the testing data and repeating this for each spectrum. The sample size (number of patients and measurements per patient) was selected with the goal of achieving two-sided 95% normal-based confidence intervals of less than ± 5% for all reported classification results, based on cross-validation.

### Data availability

The datasets generated during and/or analysed during the current study are available from the corresponding author on reasonable request.

## Electronic supplementary material

Supplemental Materials
