# Supplementary material for: A new method using Raman spectroscopy for in vivo targeted brain cancer tissue biopsy
Source: Sci Rep. 2018 Jan 29;8:1792. doi: 10.1038/s41598-018-20233-3 (PMC5788981; doi:10.1038/s41598-018-20233-3)
Supplement: Supplementary file 1 — Supplemental Materials [file 41598_2018_20233_MOESM1_ESM.docx]

A new method using Raman spectroscopy for *in vivo* targeted brain cancer tissue biopsy

Joannie Desroches^1,2^, Michael Jermyn^1,3^, Michael Pinto^1^, Fabien Picot^1^, Marie-Andrée Tremblay^1^, Sami Obaid^5^, Eric Marple^6^, Kirk Urmey^6^, Dominique Trudel^7^, Gilles Soulez^2,8^, Marie-Christine Guiot^9^, Brian C. Wilson^10^, Kevin Petrecca^4†^*, Frédéric Leblond^1,2†^*

^1^ Dept. of Engineering Physics, Polytechnique Montreal, CP 6079, Succ. Centre-Ville, Montreal, QC, H3C 3A7, Canada

^2^ Centre de Recherche du Centre Hospitalier de l’Université de Montréal, 900 rue Saint-Denis, H2X 0A9, QC, Canada

^3^ Thayer School of Engineering, Dartmouth College, 14 Engineering Dr, Hanover, NH 03755, USA

^4^ Brain Tumour Research Centre, Montreal Neurological Institute and Hospital, Dept. of Neurology and Neurosurgery, McGill University, 3801 University St., Montreal, QC, H3A 2B4, Canada

^5^ Division of Neurosurgery, Hôpital Notre-Dame du CHUM, University of Montreal, Montreal,1560 Sherbrooke E, Montreal, QC H2L 4M1, Canada

^6^ EMVision LLC, 1471 F Road, Loxahatchee, Florida, 33470, United-States

^7^ Department of Pathology, Centre Hospitalier Universitaire de Montréal, Montreal, Québec, Canada, H2X 3J4, University of Montreal Hospital Research Center (CRCHUM)

^8^ Centre hospitalier de L'Université de Montréal, Hôpital Notre-Dame-Pavillon Lachapelle, Montréal, QC, H2L 4M1, Canada

^9^ Division of Neuropathology, Department of Pathology, Montreal Neurological Institute and Hospital, McGill University, 3801 University St., Montreal, QC, H3A 2B4, Canada

^10^ University Health Network/University of Toronto, TMDT 15-314, 101 College St., Toronto, ON, M5G 1L7, Canada

^†^These authors contributed equally to this work

*Corresponding authors: [Frederic.leblond@polymtl.ca](mailto:Frederic.leblond@polymtl.ca), Kevin.petrecca@mcgill.ca

**SUPPLEMENTAL**

**Table S1**: Prominent Raman peaks. For each peak, a bootstrapping student t-test was applied for two categories (normal vs. cancer), and the corresponding p-value is provided.

| Band (cm^-1^) | Dominant | Molecular Species | Details | *p*-value |
| --- | --- | --- | --- | --- |
| 2845 | Normal | Lipids and proteins | CH_2_ symmetric stretch of lipids & CH_2_ asymmetric stretch of lipids and proteins | < 0.0001 |
| 2884 | Normal | Lipids and proteins | CH_2_ symmetric stretch of lipids & CH_2_ asymmetric stretch of lipids and proteins | < 0.0001 |
| 2930 | Cancer | Proteins and lipids | Symmetric CH_3_ stretch due primarily to protein | < 0.0001 |
| 3450 | Cancer | Water | OH stretching | 0.7968 |

**Table S2**: Comparison of the geometry of probes and acquisition parameters used in all experiments

|  | Power at tip  (mW) | Acquisition time  (s) | Fiber diameter (µm )  Illumination/  Collection | *n*  collection fibers | *n*  spectra/  measurement |
| --- | --- | --- | --- | --- | --- |
| Intraoperative probe *In vivo* in humans | 40-65 | 0.05 | 272/300 | 7 | 3 |
| Biopsy needle probe *Ex vivo* calf brain | 10 | 0.5 - 2 | 100/100 | 1 | 3 |
| Biopsy needle probe *In vivo* pig brain | 10 | 0.5 | 100/100 | 1 | 3 |
